# Clinical Outcomes of Sequential Long‐Pulsed 1060‐nm and 805‐nm Laser Treatment for Mid‐ and Lower‐Face Aging: A Single‐Arm Prospective Study

**DOI:** 10.1111/jocd.71225

**Published:** 2026-09-30

**Authors:** Li Wang, Xu Yuan, Li Liu, Lin Gao

**Affiliations:** ^1^ Department of Dermatology and Aesthetic Medicine Honghui Hospital, Xi'an Jiaotong University Xi'an China

**Keywords:** 805‐nm laser, facial rejuvenation, long‐pulsed 1060‐nm laser, skin laxity

## Abstract

**Background:**

Mid‐ and lower‐face aging involves skin laxity, textural deterioration, and contour changes. Sequential long‐pulsed 1060‐ and 805‐nm laser treatment has been proposed as a noninvasive rejuvenation approach, but evidence regarding its clinical outcomes remains limited.

**Objective:**

To assess the clinical outcomes and tolerability of sequential long‐pulsed 1060‐ and 805‐nm laser treatment for mid‐ and lower‐face aging.

**Methods:**

Twenty‐eight patients with mid‐ and lower‐face aging changes underwent three treatment sessions at 4‐week intervals using sequential long‐pulsed 1060‐ and 805‐nm diode lasers. Assessments were performed at baseline and 12 weeks after the final treatment.

**Results:**

At 12 weeks after the final treatment, significant reductions were observed in wrinkle scores, indentation index, and visible pore count, while skin elasticity improved significantly compared with baseline. GAIS assessment showed that 96.43% of patients achieved at least mild improvement, and 71.43% achieved marked or greater improvement. Treatment was well‐tolerated. No serious adverse events were observed.

**Conclusion:**

This preliminary, uncontrolled single‐arm study suggests sequential long‐pulsed 1060‐ and 805‐nm laser treatment may provide a safe, well‐tolerated noninvasive option for improving mid‐ and lower‐face aging.

## 1 Introduction

Facial aging is driven by both intrinsic aging and extrinsic environmental damage [1]. Its clinical manifestations are not limited to superficial textural changes and loss of skin elasticity but also include structural changes such as mid‐ and lower‐face soft‐tissue descent, displacement of fat pads, deepening of the nasolabial folds, formation of marionette lines, and blurring of the mandibular contour [2, 3].

Among these changes, laxity and contour alteration of the mid and lower face are considered key contributors to an aged facial appearance and represent common concerns prompting patients to seek aesthetic treatment [4]. However, in mid‐ and lower‐face rejuvenation, treatment directed solely at epidermal appearance or dermal collagen loss often fails to produce a balanced and harmonious global improvement. Therefore, combined strategies that address both deep‐tissue support and superficial‐to‐mid‐dermal skin tightening have become an important direction in noninvasive facial rejuvenation.

Long‐pulsed 1060‐ and 805‐nm diode lasers differ in tissue penetration depth and therapeutic targets. The 1060‐nm wavelength is characterized by relatively low melanin absorption and greater tissue penetration, allowing controlled heating of deeper dermal layers [5]. By comparison, the 805‐nm wavelength is expected to produce relatively more superficial dermal thermal effects, which may theoretically contribute to dermal responses associated with skin tightening [6]. On the basis of the complementary treatment depths and targets of these two wavelengths, this study used a sequential protocol combining long‐pulsed 1060‐ and 805‐nm lasers to assess the clinical outcomes and tolerability of this sequential protocol for improving signs of mid‐ and lower‐face aging.

## 2 Materials and Methods

### 2.1 Patients

This study enrolled 28 patients with mid‐ and lower‐face aging who visited the dermatology and aesthetic medicine department of a tertiary affiliated hospital between September 2025 and November 2025. The cohort included 6 men and 22 women, aged 27–56 years, with a mean age of 41.5 ± 7.8 years. Fitzpatrick skin types ranged from II to IV. The study was conducted in accordance with the Declaration of Helsinki and was approved by the Ethics Committee. All patients were fully informed and provided written informed consent.

### 2.2 Inclusion and Exclusion Criteria

Inclusion criteria were age 20–60 years; Fitzpatrick skin types II–IV; the presence of at least one investigator‐confirmed aging‐related feature of the mid or lower face, including wrinkles, skin laxity, or loss of contour definition; and a desire for noninvasive facial rejuvenation. Exclusion criteria were as follows: active or progressive facial skin diseases, such as acne, acute eczema, vitiligo, or infection; dense facial hair or a full beard; receipt of treatments that could interfere with efficacy assessment within the previous 6 months, including face‐lift surgery, radiofrequency lifting, focused ultrasound, or botulinum toxin injection; receipt of intense pulsed light, laser treatment, mesotherapy, or chemical peeling within the previous 3 months; a tendency toward keloid or hypertrophic scarring, a history of photosensitive disease, or use of photosensitizing medications during the study period; long‐term outdoor work or expected sun exposure after treatment without the ability to maintain effective photoprotection; severe systemic disease or autoimmune disease; pregnancy, lactation, or inability to use reliable contraception during the study; and any other condition that, in the investigator's judgment, made the patient unsuitable for participation.

### 2.3 Treatment Protocol

A diode laser system (Quattro; Lumenis, USA) was used. In all patients, the treatment areas included the mandibular contour, bilateral cheeks, zygomatic region, and temporal region. Treatment was performed in two steps. First, a 1060HS handpiece (1060 nm) was used with a pulse width of 400 ms, double pulse mode, and fluence of 5–6 J/cm^2^, with 350 shots delivered to each side. Skin surface temperature was monitored in real time using a thermal imaging camera (H16PRO, HIKMICRO, China) and maintained at 40°C–42°C for approximately 15 min. Mild diffuse erythema was observed in the treated area. Second, an 805‐nm XC handpiece (805 nm) was used with a pulse width of 30 ms and fluence of 12–15 J/cm^2^, with 1000 shots delivered to each side. The clinical endpoint was uniform erythema in the treatment area. After treatment, patients were advised to enhance moisturization and apply broad‐spectrum sunscreen with SPF ≥ 30 and PA ≥ ++ for effective photoprotection. Treatment was performed once every 4 weeks for a total of three sessions.

### 2.4 Outcome Measures and Assessment

Follow‐up assessments were performed at baseline, before each treatment session, 4 weeks after the final treatment (safety‐only evaluation), and 12 weeks after the final treatment. Standardized medical photographs were taken with a digital camera (EOS 80D; Canon, Japan) under uniform lighting, fixed positioning, and consistent imaging conditions.

#### 2.4.1 Objective Assessments

Antera 3D (Miravex Limited, Ireland) was used to measure wrinkle‐related facial parameters; higher scores indicated more severe wrinkles. The MPA580 skin measurement system (Courage + Khazaka electronic GmbH, Germany) was used to assess the R2 value of skin elasticity. The R2 value reflects overall skin elasticity and resistance to deformation, with values closer to 100% indicating better elasticity. The CBS Skin Analysis System (Wuhan Boshi Electronic Co. Ltd., China) was used to capture and analyze pore counts.

#### 2.4.2 Subjective Assessments

Pain intensity was evaluated immediately after each treatment session using a numerical rating scale (NRS; Table S1), and the highest score across the three sessions was used for pain‐tolerability analysis. An independent dermatologist, who was not involved in subject enrollment or treatment delivery, assessed standardized medical photographs using the Global Aesthetic Improvement Scale (GAIS; Table 2). Since GAIS grading relies on direct side‐by‐side comparison of baseline and post‐treatment images, blinding to photograph time‐points was not achievable. GAIS categories included very much improved, much improved, mildly improved, no change, and worse. Mild improvement or greater was defined as effective, whereas much improvement or greater was defined as marked improvement.

#### 2.4.3 Safety Assessment

Treatment‐emergent adverse events (TEAEs) were systematically collected throughout the study period, including immediately after each laser session, at every pre‐treatment visit, and at the 4‐ and 12‐week follow‐up after the final treatment. Adverse‐event information was obtained through investigator‐led physical examination and open‐ended patient self‐reporting. All observed or reported local cutaneous reactions, including persistent erythema, swelling, blistering, crusting, scarring, dyspigmentation, folliculitis, and hair‐related abnormalities, were documented. Each adverse‐event was characterized with respect to onset time, duration, maximum severity (mild/moderate/severe), any interventions administered, and clinical resolution status. Severity grading of adverse events followed standard dermatologic laser research criteria: mild (well‐tolerated, no interference with daily activities); moderate (partial interference with daily activities); severe (marked discomfort, substantial impairment of daily activities).

### 2.5 Statistical Analysis

All data were processed and analyzed using IBM SPSS Statistics version 25.0. Given the exploratory, preliminary single‐arm design of this study, no formal sample‐size power calculation was performed for efficacy endpoints. The primary outcome was defined as the change in wrinkle score measured by Antera 3D at 12 weeks after the final treatment compared with baseline. Secondary outcomes included changes in indentation index, skin elasticity R2 value, visible pore count, GAIS categorical distribution, and NRS pain scores. Follow‐up assessments were scheduled at baseline, before each treatment session, 4 and 12 weeks after the final treatment. The 4‐week time point after final treatment was designated for safety observation only and was not pre‐specified for efficacy statistical analysis. From a clinical perspective, 12 weeks after the final treatment was selected as the primary efficacy time point to allow sufficient time for clinical aesthetic changes to stabilize after laser intervention, compared with week 4. Therefore, only baseline and 12‐week post‐treatment efficacy outcomes are reported in the present manuscript. The paired‐samples *t*‐test was used to analyze changes in continuous variables from baseline to 12 weeks after the final treatment. Categorical variables were expressed as counts and percentages. A *p* value < 0.05 was considered statistically significant.

## 3 Results

### 3.1 Baseline Demographic Characteristics

A total of 28 patients with aging‐related changes of the mid and lower face were enrolled, including 6 men and 22 women. The patients were 27–56 years of age, with a mean age of 41.5 ± 7.8 years. Fitzpatrick skin types were Type II in 8 patients, Type III in 16 patients, and Type IV in 4 patients (Table 1). All patients completed three treatment sessions and the 12‐week follow‐up after the final treatment.

**TABLE 1:** Baseline demographic characteristics.

Variables	Value
Number of patients	28
Age, years, mean ± SD	41.5 ± 7.8
Age range, years	27–56
Sex, *n* (%)	
Female	22 (78.6%)
Male	6 (21.4%)
Fitzpatrick skin type, *n* (%)	
Type II	8 (28.6%)
Type III	16 (57.1%)
Type IV	4 (14.3%)

### 3.2 Subjective Assessment

Most patients showed varying degrees of improvement in mid‐ and lower‐face laxity and wrinkles. GAIS analysis showed an overall response rate of 96.43% (27/28) and a marked improvement rate of 71.43% (20/28), with 17.86% (5/28) of patients rated as very much improved (Table 2). Representative cases demonstrated visible improvement in nasolabial folds, marionette lines, and mandibular contour (Figure 1), while image‐derived analysis showed reductions in visible pore distribution and counts (Figure 2). These findings suggest that the treatment protocol was associated with visible global aesthetic improvement in most patients. During treatment, NRS pain scores were 1 in 14 patients (50.00%), 2 in 12 patients (42.86%), and 3 in 2 patients (7.14%) (Table S2). Overall, 92.86% of patients reported pain scores of 2 or lower, indicating mild pain and good treatment tolerability.

**TABLE 2:** Global aesthetic improvement scale (GAIS).

GAIS	*n*	%
Very much improved	5	17.86
Much improved	15	53.57
Mildly improved	7	25.00
No change	1	3.57
Worse	0	0.00
Total	28	100.00

**FIGURE 1:**
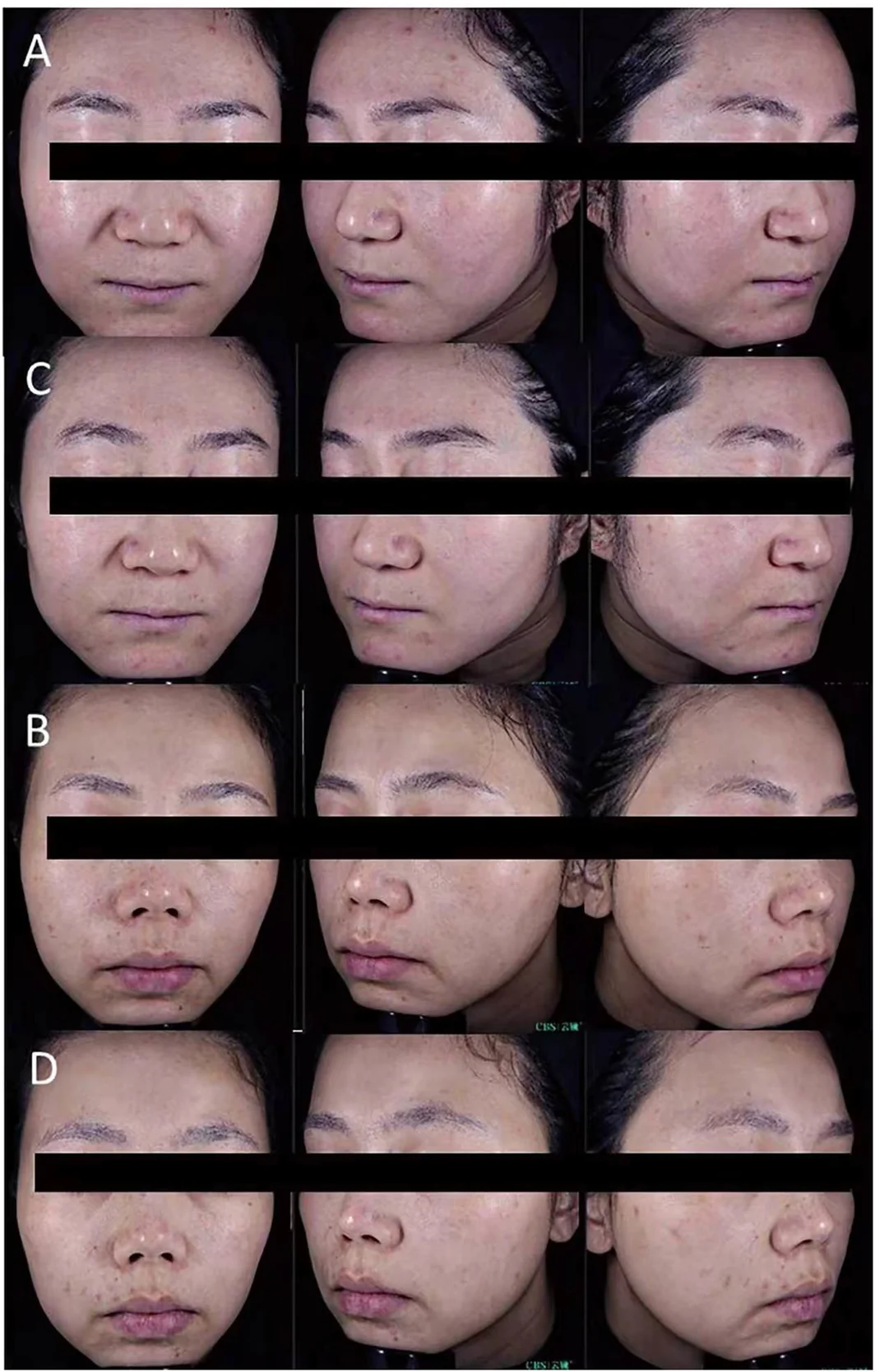
CBS skin analysis system images of two representative patients before and at 12 weeks after the final treatment. (A and B) Before treatment; (C and D) 12 weeks after the final treatment.

**FIGURE 2:**
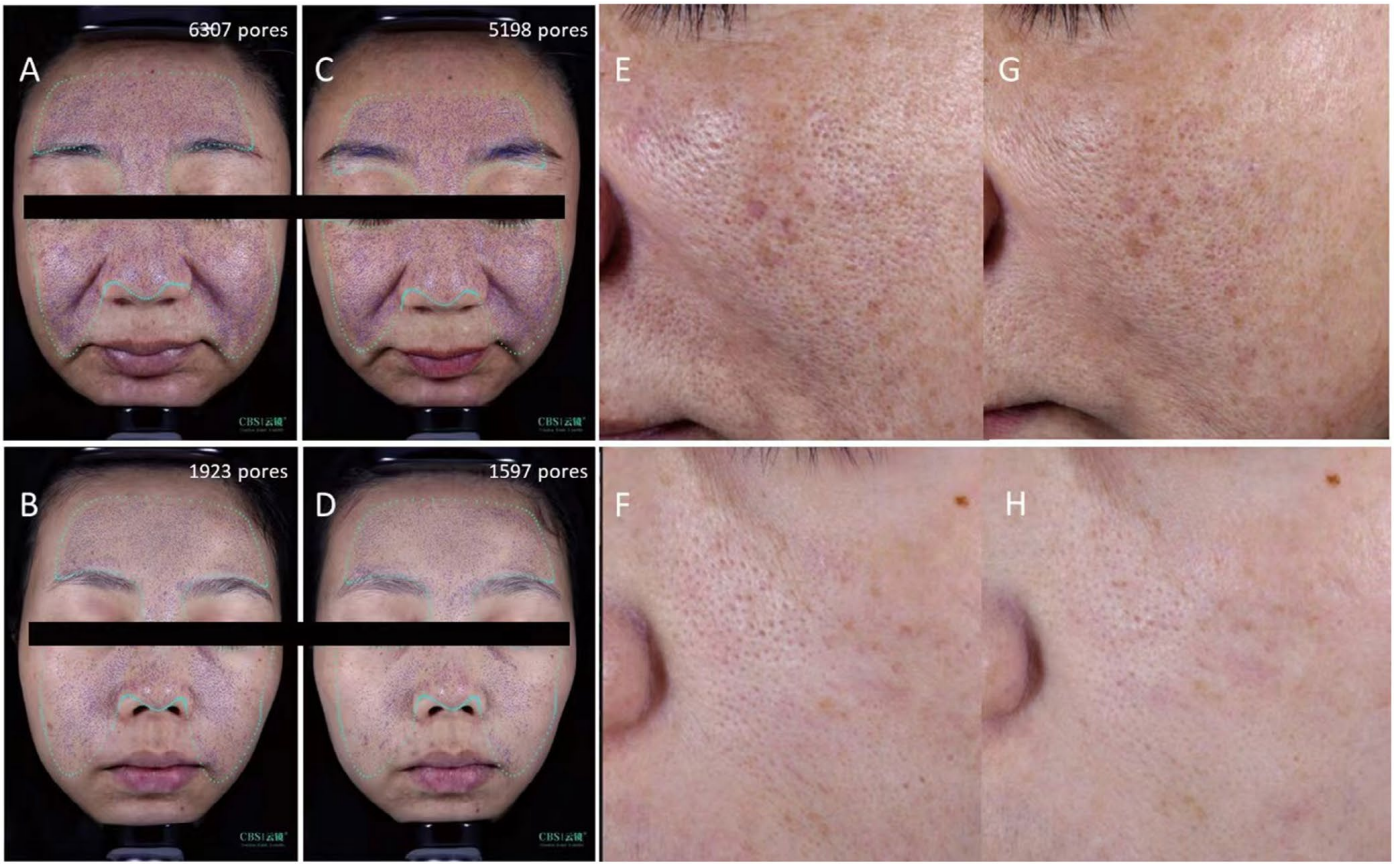
Visible pore distribution and count in two representative patients before treatment and at 12 weeks after the final treatment. (A and B) Before treatment; (C and D) 12 weeks after the final treatment; (E and F) enlarged views of the left cheek before treatment; (G and H) enlarged views of the left cheek at 12 weeks after the final treatment.

### 3.3 Objective Assessment

Of all planned follow‐up time‐points, only baseline and 12‐week post‐final‐treatment efficacy data are presented below; the 4‐week follow‐up was performed solely for safety surveillance. Antera 3D and MPA580 assessments showed that, compared with baseline, wrinkle score and indentation index were significantly decreased at 12 weeks after the final treatment, whereas the R2 value of skin elasticity was significantly increased (all *p* < 0.001) (Table 3, Figure 3). Analysis using the CBS Skin Analysis System showed a significant reduction in image‐derived visible pore count compared with baseline (*p* < 0.001) (Table 3, Figure 2). These results suggest that combined long‐pulsed 1060‐ and 805‐nm laser treatment may improve wrinkles, localized contour depression, and skin elasticity in the mid and lower face.

**TABLE 3:** Changes in objective skin parameters from baseline to 12 weeks after the final treatment (mean ± SD).

Parameter	Baseline	12 weeks after final treatment	*t*	*p*
Wrinkle score	42.18 ± 10.53	35.82 ± 10.43	9.826	< 0.001
Indentation index	46.32 ± 9.91	40.31 ± 9.41	10.579	< 0.001
Elasticity index R2 (%)	58.38 ± 9.72	77.34 ± 10.55	−7.28	< 0.001
Visible pore count, *n*	4564.43 ± 1709.03	3353.46 ± 1531.02	10.581	< 0.001

*Note:* Paired‐samples *t*‐test was used. *p* < 0.05 was considered statistically significant.

**FIGURE 3:**
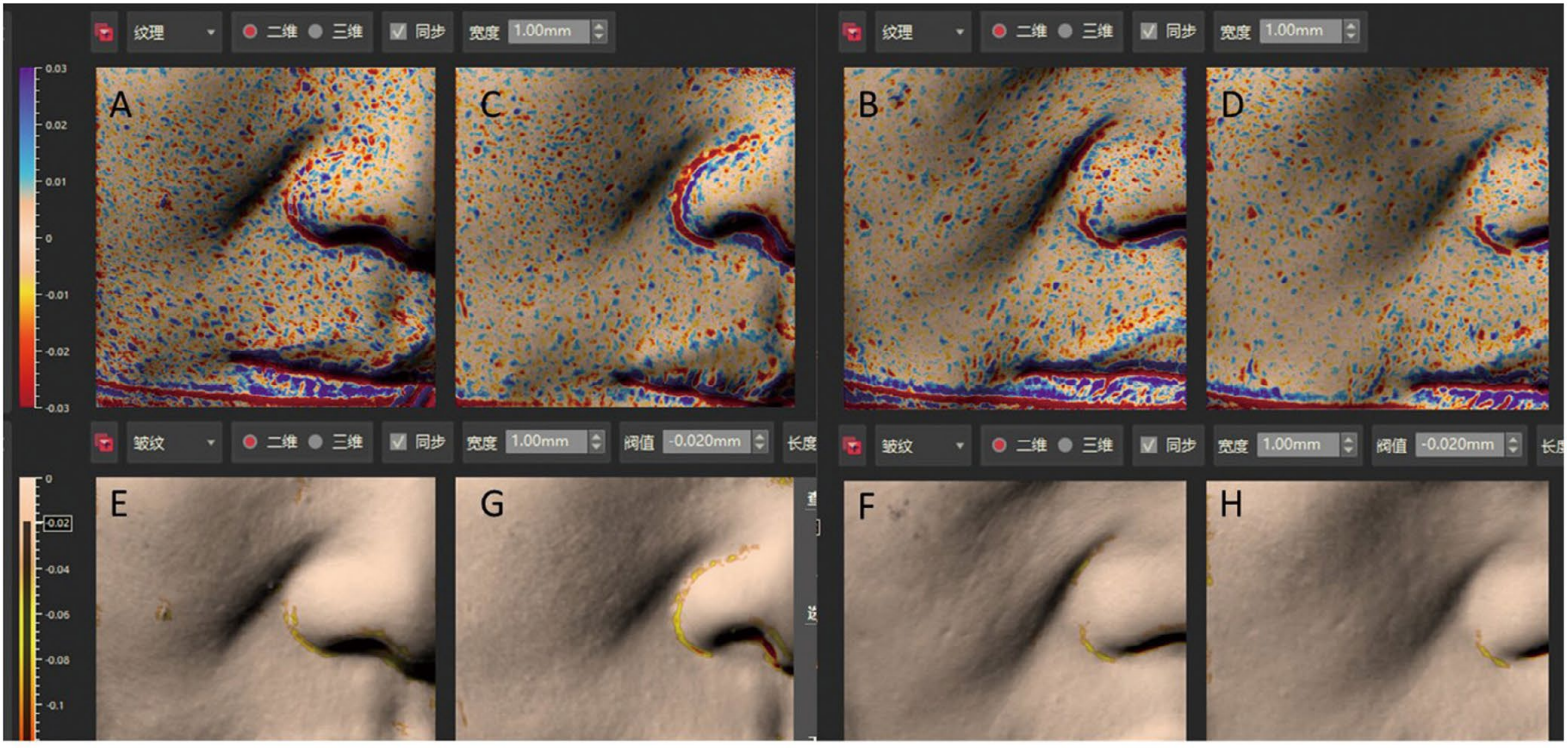
Antera 3D assessments of two representative patients before treatment and at 12 weeks after the final treatment. (A and B) Texture before treatment; (C and D) texture changes at 12 weeks after the final treatment; (E and F) wrinkle before treatment; (G and H) wrinkle changes at 12 weeks after the final treatment.

### 3.4 Safety Assessment

No serious adverse events, including blistering, scarring, and pigmentary changes occurred in any patient. Four patients developed persistent mild erythema and edema after treatment, all of which resolved spontaneously within 2 days. During follow‐up, no abnormal hair growth, hair reduction, folliculitis, or other hair‐related adverse events were observed in the treated areas.

## 4 Discussion

The present study showed that sequential treatment with long‐pulsed 1060‐ and 805‐nm lasers may improve aging‐related changes of the mid and lower face to a certain extent. At 12 weeks after the final treatment, wrinkle‐related scores, indentation index, and visible pore count decreased compared with baseline, whereas the R2 elasticity parameter increased. GAIS assessment also indicated that most patients achieved at least mild overall improvement. Pain during treatment was low, and adverse reactions were mild and transient, suggesting that this combined protocol has good tolerability and preliminary clinical feasibility.

Aging of the mid and lower face is not simply a consequence of epidermal or dermal photoaging; rather, it is a complex process involving the skin, subcutaneous fat, retaining ligament support, and gravitational soft‐tissue changes [7]. Clinically, patients often present with a combination of deepened nasolabial folds, marionette lines, cheek soft‐tissue laxity, blurred mandibular contours, and rough skin texture [8]. Therefore, treatment strategies for mid‐ and lower‐face rejuvenation that focus only on superficial wrinkles or skin texture may be insufficient to achieve meaningful global improvement. By contrast, multilayered, minimally traumatic, and repeatable energy‐based approaches may better meet the treatment needs of patients with aging changes in this region.

The rationale for combining 1060‐ and 805‐nm wavelengths was to generate a layered thermal response rather than simply increase total fluence. Experimental data on wavelength‐dependent light–tissue interactions suggest that wavelengths around 1064 nm have relatively lower scattering and lower absorption by melanin and hemoglobin than shorter near‐infrared wavelengths, supporting greater tissue penetration. In this context, the 1060‐nm wavelength may provide relatively deeper dermal thermal stimulation, whereas the 805‐nm wavelength may offer comparatively more superficial near‐infrared photothermal effects [6]. We hypothesize that this layered thermal effect may drive clinical improvement via dermal thermal responses. However, neocollagenesis, deep‐tissue remodeling, and structural soft‐tissue lifting were not directly evaluated in the present study, and such mechanistic explanations remain speculative.

The improvements observed in this study were not limited to a single parameter. Decreases in wrinkle score and indentation index suggested attenuation of groove‐like wrinkles and contour depression in the mid and lower face, while the increase in R2 reflected improved mechanical elasticity of the skin. The reduction in image‐derived visible pore count further suggested improvement in skin surface texture and smoothness. Together, these changes likely contributed to the observed improvement in GAIS scores. The clinical relevance of this sequential protocol may therefore be related less to isolated wrinkle reduction or tightening than to concurrent, modest improvements across contour‐related features and skin quality. This pattern is consistent with the clinical complexity of mid‐ and lower‐face aging, in which structural laxity, groove‐like depressions, and textural changes often coexist. However, because histologic, ultrasound, or other structural assessments were not performed, the precise tissue‐level mechanisms underlying these changes remain uncertain and warrant further investigation.

Regarding safety, pain scores were low during treatment, and no serious adverse events were observed. The adverse reactions recorded were mainly transient erythema, mild swelling, or a sensation of heat, all of which resolved spontaneously. It should be noted that melanin absorption remains relevant at the 805‐nm wavelength, and long‐pulsed 1060‐nm laser treatment may also generate thermal effects in deeper tissues. Therefore, combined use of these wavelengths could theoretically affect follicular structures or hair growth. For this reason, patients with a full beard or obvious facial hair were excluded at enrollment. Although no hair reduction, folliculitis, abnormal hair growth, or other hair‐related adverse events were observed during follow‐up, these findings mainly apply to patients without prominent facial hair. In addition, given that this study lacks a single‐wavelength intervention arm and thus cannot distinguish the respective contributions of the two lasers, together with the relatively limited number of treatment‐related assessments and follow‐up restricted to 12 weeks after the final treatment, future investigations should adopt larger sample sizes and randomized controlled designs comparing standalone 1060‐nm laser, standalone 805‐nm laser, and this sequential combined protocol to clarify whether combined treatment provides incremental benefits relative to single‐wavelength modalities.

Another important limitation is the absence of histopathological or ultrasound‐based tissue assessment. Improvements in wrinkles, indentation index, skin elasticity, and contour appearance were captured only by non‐invasive surface imaging and photographic evaluation; therefore, we cannot draw definitive conclusions regarding underlying biological processes such as neocollagenesis, deep‐dermal remodeling, or structural soft‐tissue lifting. Inferences about tissue‐level mechanisms should be interpreted with caution. Moreover, our cohort was limited to Fitzpatrick skin types II–IV and excluded subjects with prominent facial hair, which restricts generalization to other skin types or patient populations.

Additionally, efficacy outcomes were only evaluated at 12 weeks after final treatment. The 4‐week follow‐up was limited to safety monitoring rather than efficacy assessment, which prevents us from capturing early dynamic changes in skin parameters after laser intervention.

In conclusion, sequential long‐pulsed 1060‐ and 805‐nm laser treatment was associated with measurable improvements in wrinkles, indentation appearance, visible pore count, and skin elasticity in patients with mid‐ and lower‐face aging. The treatment was generally well‐tolerated, with no serious adverse events observed. These preliminary findings suggest that this approach may be a noninvasive option for selected patients. However, larger randomized controlled studies with longer follow‐up are needed to confirm its efficacy, durability, optimal parameters, and long‐term safety.

## Author Contributions

**Li Wang:** writing – original draft, formal analysis, visualization. **Xu Yuan:** writing – review and editing, conceptualization. **Li Liu:** data curation. **Lin Gao:** supervision. All authors have read and approved the final manuscript.

## Funding

The authors have nothing to report.

## Ethics Statement

The study was conducted in accordance with the Declaration of Helsinki and was approved by the Ethics Committee of Honghui Hospital, Xi'an Jiaotong University (approval No. 2025‐Ethics Opinion‐KY‐187‐01).

## Consent

Written informed consent was obtained from all patients for publication of clinical data and accompanying images.

## Conflicts of Interest

The authors declare no conflicts of interest.

## Supporting information


**Table S1:** Numerical rating scale (NRS) for pain assessment.
**Table S2:** Distribution of NRS pain scores (highest score across three treatment sessions).

## Data Availability

The raw clinical dataset generated and analyzed during the current study is not publicly available due to patient‐privacy‐related ethical restrictions. De‐identified aggregated data may be available from the corresponding author upon reasonable request and institutional ethics approval.
