# Supplementary material for: Clinical Outcomes of Sequential Long‐Pulsed 1060‐nm and 805‐nm Laser Treatment for Mid‐ and Lower‐Face Aging: A Single‐Arm Prospective Study
Source: J Cosmet Dermatol. 2026 Sep 30;25(10):e71225. doi: 10.1111/jocd.71225 (PMC13627275; doi:10.1111/jocd.71225)
Supplement: Supplementary file 1 [file JOCD-25-e71225-s001.docx]

**Table S1. Numerical Rating Scale (NRS) for Pain Assessment**

| **Item** | **Description** |
| --- | --- |
| Score range | 0–10 |
| Score 0 | No pain |
| Score 10 | Worst imaginable pain |
| Assessment | Self-reported by the subject |

*Note: Pain intensity was evaluated immediately after each treatment session using the NRS. The highest score across the three treatment sessions was used for pain-tolerability analysis.*

**Table S2. Distribution of NRS Pain Scores (Highest Score Across Three Treatment Sessions)**

| **NRS Score** | **n** | **%** |
| --- | --- | --- |
| 1 | 14 | 50.00 |
| 2 | 12 | 42.86 |
| 3 | 2 | 7.14 |
| Total | 28 | 100.00 |

*Note: Overall, 92.86% of patients reported NRS pain scores of 2 or lower, indicating mild pain and good treatment tolerability. No patient reported a score greater than 3.*
